# Deformation Mechanism of Aluminum, Copper, and Gold in Nanoimprint Lithography Using Molecular Dynamics Simulation

**DOI:** 10.3390/nano13243104

**Published:** 2023-12-08

**Authors:** Abhaysinh Gaikwad, Michael Olowe, Salil Desai

**Affiliations:** 1 Center for Excellence in Product Design and Advanced Manufacturing, North Carolina A & T State University, Greensboro, NC 27411, USA; ahgaikwa@aggies.ncat.edu (A.G.); msolowe@aggies.ncat.edu (M.O.); 2 Department of Industrial & Systems Engineering, North Carolina A & T State University, Greensboro, NC 27411, USA

**Keywords:** deformation mechanism, polyhedral template matching, lattice dislocations, nanoimprint lithography, molecular dynamics, spring-back

## Abstract

Material deformation during nanoimprinting of aluminum (Al), copper (Cu), and gold (Au) was explored through molecular dynamics simulations. A comparative understanding of the deformation behavior of three substrate materials important for design and high-resolution pattern transfer was highlighted. In this study, we analyzed three metrics, including von Mises stresses, lattice deformation, and spring-back for the chosen materials. Of the three materials, the highest average von Mises stress of 7.80 MPa was recorded for copper, while the lowest value of 4.68 MPa was computed for the gold substrate. Relatively higher von Mises stress was observed for all three materials during the mold penetration stages; however, there was a significant reduction during the mold relaxation and retrieval stages. The Polyhedral Template Matching (PTM) method was adopted for studying the lattice dislocation of the materials. Predominantly Body-Centered Cubic (BCC) structures were observed during the deformation process and the materials regained more than 50% of their original Face-Centered Cubic (FCC) structures after mold retrieval. Gold had the lowest vertical spring-back at 6.54%, whereas aluminum had the highest average spring-back at 24.5%. Of the three materials, aluminum had the lowest imprint quality due to its irregular imprint geometry and low indentation depth after the NIL process. The findings of this research lay a foundation for the design and manufacture of Nanoimprint Lithography (NIL) molds for different applications while ensuring that the replicated structures meet the desired specifications and quality standards.

## 1. Introduction

Nanoimprint Lithography has become one of the advanced nanofabrication methods used to create high-quality nanopatterns. The principle of NIL is to transfer patterns on the mold by physical deformation of polymer resist by pressing the mold into resist [1,2,3,4]. In traditional photolithography, there is no physical contact between the mold and the resist; however, the photolithography process is constrained by the wavelength of the exposed UV light for fabricating nanoscale structures and the secondary processes such as etching and metallization [5,6]. The NIL, on the other hand, is a single-step process and provides high throughput and high replication at low cost [7]. In the early stages, NIL was predominantly used for nanopatterning of polymers and thermoplastic resist materials [8,9,10,11]. Since the metals featuring different nanostructures and patterns have found wide applications in semiconductor, optoelectronics, and biological applications, the principle of NIL has been applied for direct metal nanopatterning [12,13].

In direct metal nanoimprinting [14,15,16,17], the mold with nanopatterns is pressed against the metal substrate to replicate the nanopatterns by physical deformation. The mechanical, electrical, and optical properties of the metal depend on the plastic deformation of the metal substrate [15]. Therefore, it is crucial to understand the deformation mechanism of the metal substrate during nanoimprinting process. The effect of different NIL process parameters on the imprint quality has been evaluated experimentally. The NIL process necessitates the use of specialized equipment, a controlled clean room, and precise substrate preparation to achieve ultra-high resolution. Therefore, experimental analysis of the deformation behavior of metals can be cost-prohibitive and time-consuming, necessitating the development of simulation models to investigate the NIL process. Moreover, understanding the dislocation and spring-back effects can present significant challenges solely with experimental analysis [7,8,18]. The finite element method (FEM) has been applied to study the cavity-filling process and induced stresses in the nanoimprint lithography process for a polymer [19]. Yasuda and his team employed FEM to investigate the material deformation during the NIL process for metal [20]. The simulation results showed the variation of pressure acting on the mold with respect to mold position during nanoimprinting of glass. The FEM characterizes the NIL process at the continuum scale but does not accurately explain the atomistic phenomenon. Molecular Dynamics (MD) has proven to be an effective tool to study material deformation at the atomic level [21]. MD simulations assist in understanding the atomistic motions and conformational changes in the substrate.

Various authors have investigated nanoimprinting of metals such as copper, aluminum, gold, metallic glass, silicon dioxide, and copper-nickel alloys through molecular dynamics simulations [22,23,24,25,26,27,28]. The simulation studies have shown the effect of temperature on punch force and internal energy, the effect of void defects on force, and the mechanical behavior of thin metal layers [22,26,29]. The simulation studies on gold and copper have shown the effect of nanoimprinting depth on the dislocation dynamics and final pattern transfer [26]. Metals such as aluminum, copper, and gold are attractive candidates for nanoimprinting because of their wide use for industry applications due to their excellent corrosion resistance, superior fabricability, chemical stability, and electrical and thermal properties [13]. Al, Cu, and Au have widespread applications such as solid-state devices in the electronic industry, bio-chem sensors, and miniaturized devices in clinical research [12]. In the past, our group has revealed the relation between imprint depth and the spring-back phenomenon and the application of artificial intelligence algorithms in nanoimprint lithography [21,30,31,32]. Further, we have investigated the effect of varying temperature, mold geometry, and mold velocity on the von Mises stress, contact pressure, and lattice dislocation of the gold substrate using molecular dynamics simulations [33,34,35].

Understanding the material deformation mechanism in the nanoimprinting of metals such as Al, Cu, and Au is critical for high-resolution nanopatterning [23,24]. However, the comparative investigation of the atomistic deformation mechanism in nanoimprinting of Al, Cu, and Au has yet to be conducted. In this paper, the material deformation behavior of Al, Cu, and Au during the nanoimprinting process is presented to reveal the spring-back phenomenon and its effect on the accuracy of final pattern transfer. Moreover, the lattice dislocations and recovery of lattice structures after the demolding phase of nanoimprinting are also explored in detail. The imprints were simulated at room temperature (293 K) for all the materials. The mold velocity and aspect ratio of the mold profile were constant for all the materials. This research provides insight into the deformation mechanism and its effect on the accuracy of the final pattern transfer after nanoimprinting of Al, Cu, and Au.

## 2. Materials and Methods

In this research, molecular dynamics simulations were performed using Large-scale Atomic/Molecular Massively Parallel Simulator (LAMMPS) open-source code [36]. LAMMPS is well-known for its robustness [37,38] for parallel computing and compatibility with most force fields. The Open Visualization Tool (OVITO) and Visual Molecular Dynamics (VMD) platforms were used to visualize the simulations and analyze the results [39,40]. The simulations were run on a 64-bit Linux Fedora 27 system with a Graphical Processing Unit (GPU) board (2 NVIDIA K40) with 2880 cores.

Typically, feature sizes in NIL range from 5 nm to over 100 nm depending on the application intent and several factors, such as the resist properties, the processing conditions, and the expected resolution of the template. Pandey et al. [41] demonstrated nanopatterning of a thermal resist on a lens surface and direct nanoimprinting of chalcogenide glass in the order of 10 nm feature sizes. Furthermore, nanoimprint-directed self-assembly (DSA), a high-resolution nanofabrication technique, has been employed by Park et al. [42] while investigating the potential of nanoimprint molds for efficiently templating the assembly of block copolymers (BCPs) within the sub-10 nm scale. Kuo et al. [43] have also demonstrated how sub-10 nm metal nanoparticles have been patterned using the technique of nanosphere lithography and NIL. Ito et al. [44] also achieved sub-15 nm patterning through a UV-assisted NIL process using a diacrylate silica mold with varying hole diameters of 7 nm, 15 nm, and 20 nm. In the work of Debbie Morecroft et al. [45], hydrogen silsesquioxane (HSQ) patterns were manufactured as a negative electron beam resist using helium electron beam lithography (EBL) in the region of sub-15 nm nodes. In similar research conducted by Peroz et al. [46], a sub-15 nm wide Al_2_O_3_-coated HSQ mold was used to create sub-10 nm patterns on a hard substrate. In our research, the Al, Cu, and Au substrates were 10 nm × 8 nm × 6 nm in dimensions and had 30,000, 42,840, and 30,000 atoms, respectively. The silicon mold was composed of 5454 atoms in total. The height and width of the mold tooth was 3.5 nm, which represents an aspect ratio of 1. Periodic boundary conditions were applied in both x and y directions. The bottom three layers of substrate atoms were fixed to ensure the rigidity of the process. The simulations were conducted at a mold velocity of 50 m/s for a time step of 1 femtosecond. The nanoimprinting process was simulated at room temperature (293 K) for Al, Cu, and Au. The simulations were performed in the microcanonical (NVE) ensemble, and a direct rescaling thermostat was applied to control the temperature. The velocity-verlet integrator was employed to run the simulation. The Modified Embedded Atom Method (MEAM) potential was used to simulate atomic force interactions between the mold and substrate, which is defined by Equation (1) [47],

(1) $$E=\sum_{i}\left\{F_{i}\left(\bar{\rho_{i}}\right)+\frac{1}{2}\sum_{i\neq j}\varphi_{ij}(r_{ij})\right\}$$

where, *F_i_* is energy to embed an atom of type *i* into the background electron density at site *i*, *ρ_i_* is the electron density, and *φ_ij_* is a pair potential interaction between atoms *i* and *j* whose separation is given by *r_ij_*. In Table 1, α^0^ is the exponential decay factor for the universal equation of state; β^0^, β^1^, and β^3^ are the exponential decay factors for the atomic electron densities; t^(1)^, t^(2)^, and t^(3)^ are the weighting parameters for the atomic electron densities. The material properties and MEAM potential are also given in Table 1.

At ambient conditions, the silicon mold gradually descends into the substrate, inducing atomic-scale displacement. During the molding stage, the mold fully embeds itself within the substrate material, initiating pattern formation. Subsequently, the mold is statically held in position during the relaxation stage. At the final stage, the silicon mold is extracted from the substrate, leaving an imprint etched onto its surface. For the nanoimprint lithography process, the mold and demolding stages affect the accuracy of the final geometry. Therefore, it is important to understand the dynamics of the two critical phases. The six pressure tensors were computed and recorded for each of the substrates, and the von Mises stresses were calculated using Equation (2).

(2) $$F_{vM}=\surd \frac{{{((P}_{xx}-P_{yy})}^{2}+{{(P}_{yy}-P_{zz})}^{2}+{{(P}_{zz}-P_{xx})}^{2}+6\times ({P_{xy}}^{2}+{P_{xz}}^{2}+{P_{yz}}^{2}))}{2}$$

The spring-back measure was implemented to quantify the imprint quality based on surface dimensions at the molding and demolding stages. A positive spring-back indicated that the substrate dimensions have reduced or contracted in respective directions; meanwhile, a negative spring-back indicated the imprinted cavity expansion. The imprint quality is considered high when the variation of spring-back is minimal in either or both directions. The effect of mold profile on the imprint quality was determined using a quantitative measure of spring back phenomenon. Percentage (%) spring back was calculated by:

(3) $$\%\;Spring\;back=\frac{L_{mold}-L_{demold}}{L_{mold}}\times 100$$

where, % *Spring back* is the spring back value in the horizontal or vertical direction, *L_mold_* is the initial dimension during molding, and *L_demold_* is the final dimension after demolding, respectively.

## 3. Results and Discussions

The behavior of the three chosen materials, namely aluminum, copper, and gold, was investigated and compared across all stages of the nanoimprint lithography process. The findings provided useful insights into their plastic deformation mechanisms after indentation, including the quality of nanoimprint patterns transferred from the silicon mold to the substrate materials. Furthermore, the dislocation mechanism of localized structures in these materials was critically studied using the Polyhedral Template Matching (PTM) method. The lattice structural changes at the molding and mold retrieval stages (demolding) were compared. Additionally, the spring-back for each material was examined and computed, highlighting its significance in designing patterns for various applications.

The imprinting was carried out at room temperature because the three materials used (aluminum, copper, and gold) all have different linear expansivity coefficients. Imprinting each material with silicon mold at room temperature will help to minimize the mismatch between the mold and the substrate materials, reducing the chance of misalignment of the imprinted patterns during indentation.

### 3.1. Variation in Von Mises Stress during the NIL Process

**Figure 1:**
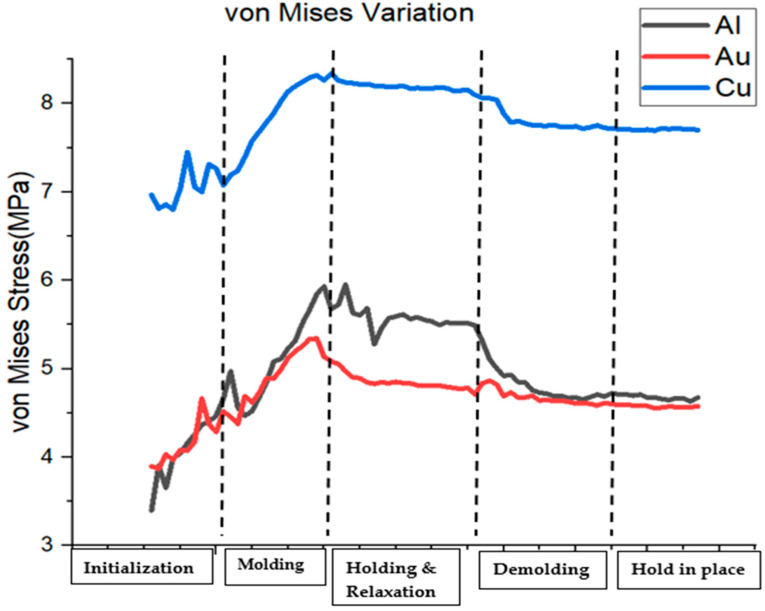
NIL stages and von Mises stress patterns for the three materials.

This section presents a comparative study of the stress patterns of aluminum, copper, and gold substrates under deformation. Figure 1 shows the variation of von Mises stress across different stages of the NIL process for each material. The highest average von Mises stress was recorded for each material towards the end of the molding stage, and the lowest von Mises stress was seen when the silicon mold was disengaged from the substrate (demolding). The theoretical explanation for this behavior is that during molding, the substrate material undergoes a large amount of deformation, which causes the displacement of atoms and results in an imbalance between the attractive and repulsive forces between them. The highest and lowest von Mises stress values of 8.34 MPa and 6.80 MPa were recorded for copper substrate, 5.95 MPa and 3.40 MPa for aluminum substrate, and 5.34 MPa and 3.87 MPa for gold substrate, respectively. Simulation results showed that copper substrate had the highest average von Mises stress value of 7.80 MPa, while the lowest average von Mises stress of 4.68 MPa was recorded for gold. Gold exhibited lower von Mises stress than copper as a result of its higher malleability. Malleability is the ability of a material to undergo plastic deformation under high strain rates. High malleability indicates that a material can undergo severe plastic deformation under complex loading conditions without experiencing fragmentation. In addition, aluminum and gold had lower ultimate tensile strengths of 90 MPa and 120 MPa, respectively, as compared to 210 MPa for copper. The differences in the ultimate tensile strengths of these three materials are reflected systematically with varying von Mises stresses during the deformation behavior. Thus, Cu had the highest von Mises stresses throughout the nanoimprinting stages, followed by aluminum and gold, as depicted in Figure 1. At the holding and relaxation stages, the von Mises stress levels were seen to be relatively stable for all three materials. Interestingly, the stress distribution patterns of copper and gold were similar at the beginning of the molding stage and at the demolding stage of the nanoimprint lithography process. It may be noted that very high von Mises stress levels may reduce imprint pattern transfer fidelity and final imprint resolution due to the formation of cracks and defects.

**Figure 2:**
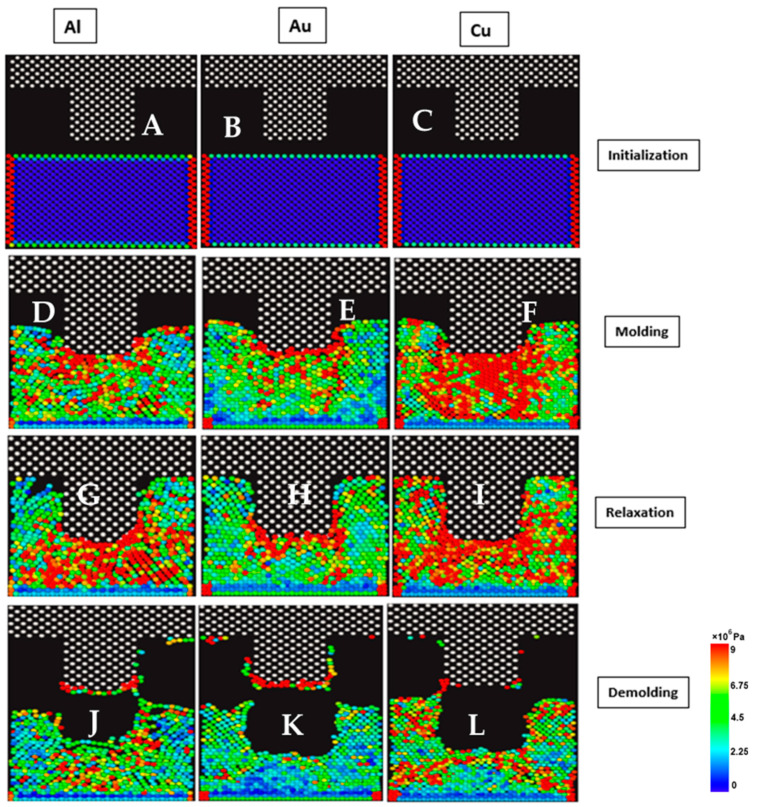
von Mises Stress profile for Al, Au, and Cu at different NIL stages. (**A**–**C**) von Mises stress pattern before the indentation, (**D**–**F**) von Mises stress pattern during the molding stage. (**G**–**I**) von Mises stress pattern at the relaxation stage. (**J**–**L**) von Mises stress pattern at the demolding stage.

The visualization results of the von Mises stress are presented in Figure 2 below for gold, aluminum, and copper substrates. As seen in the image, the high-density “red zones” around neighboring clusters of atoms indicate regions where the stress levels were at the highest. Before the mold penetration stage, the three substrate materials exhibited relatively lower average von Mises stress values below 2.25 MPa, as indicated by the color scale in Figure 2, where the “blue” color corresponds to these lower stress levels. This scale uses different colors to represent varying stress levels. Higher stress levels were observed when the silicon mold made contact with the substrate and was slowly depressed into the substrate material (molding stage). A reduction in the von Mises stresses was observed during the relaxation stage for all three materials. This is due to the realignment of substrate atoms within the materials during the holding phase. Further, the localized stress was relatively lower at the demolding stages for the individual substrate materials. These results were consistent with the outcome of the von Mises stress patterns presented in Figure 1. The von Mises stress profile of copper showed that there were higher stress values for copper at the molding stage as compared to gold and aluminum. After demolding, some atoms were seen sticking to the surface of the silicon mold due to high mold-substrate interactions during mold indentation. The number of atoms that bonded to the silicon molds was not significant for the three materials. Understanding the stress distribution and behavior of materials at different stages in the NIL process is crucial for optimizing the fabrication process and ensuring the desired patterns are realized without compromising material integrity.

### 3.2. Influence of Dislocation Phenomenon on the Deformation

**Figure 3:**
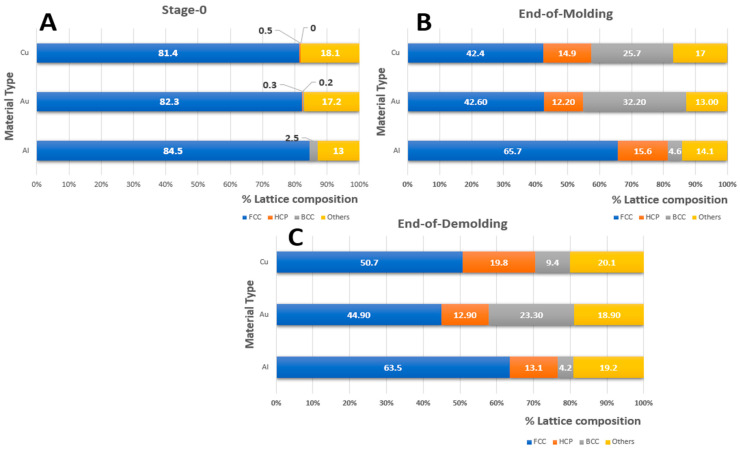
Lattice distribution for the three materials at the initial stage, End-of-Molding & End-of-Demolding. (**A**) Crystal lattice distribution for at the initial stage. (**B**) Crystal lattice distribution at the End-of-Molding. (**C**) Crystal lattice distribution at the End-of-Demolding.

The dislocation behavior of the three substrate materials was also investigated using the Polyhedral Template Matching (PTM) method. Figure 3 shows the percentage composition of each lattice structure retained in the materials during the molding and demolding stages. When the materials were indented and underwent plastic deformation, some of the lattice structure, predominantly FCC (81% for Cu, 82% for Au, and 85% for Al), of the substrate materials were observed to change to BCC, Hexagonal Close-Packed (HCP) and other structures (such as icosahedron). Comparing the lattice structures at the EOM (End-of-Molding) and EOD (End-of-Demolding), the FCC structure of copper increased significantly from 42% to 51%, and that of gold increased slightly from 43% to 45%. Also, there was an observed decrease in the FCC structure for aluminum from 66% to 64%. The transformation can be credited to the Bain path, which consists of the dilation of the FCC structures in the x and y directions due to the motion of atoms away from the mold insertion region. At the end of demolding, the HCP structure for both copper and gold increased from 15% to 20% and 12% to 13%, respectively, while that of aluminum dropped by 3% from 16% to 13%. There was a significant drop in the BCC structure from 26% to 9% for copper. However, there was little to no difference recorded in the BCC structure for Aluminum during both stages. It can be inferred from these results that the copper substrate material regained some of its lattice structure after the demolding stage and can be considered to have retained its mechanical properties (such as ductility and formability) even under plastic deformation.

**Figure 4:**
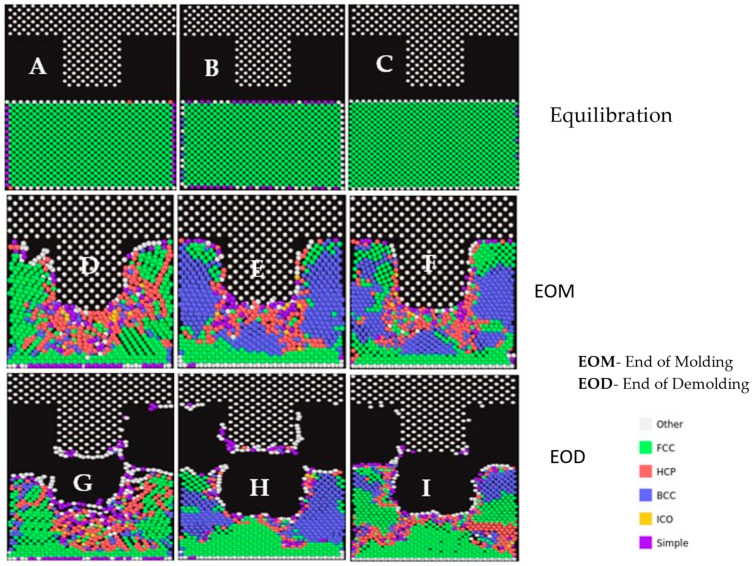
Polyhedral Template Matching (PTM) profiles for the three materials at Equilibration, End-of-Molding and End-of-Demolding Stages. (**A**–**C**) PTM profile at equilibration. (**D**–**F**) PTM profile at EOM. (**G**–**I**) PTM profile at EOD.

Figure 4 revealed the lattice structures at the initial stage, end of molding, and demolding stages of the NIL process for all three materials. The image was obtained by slicing the substrate materials during simulation at a defined width in the Y-plane at the respective stages. From the simulation results, prior to the molding stage, the crystal lattice structure of the three materials was largely FCC, as presented in Figure 3 above. From Figure 4, the gold substrate had the cleanest imprint after the complete withdrawal of the silicon mold. The imprint within copper was similar to gold, with minor deformation at the top edges of the substrate material. However, the imprint within the Al material had the lowest imprint depth with significant deformation. Al, Cu, and Au have FCC structures and are malleable. Malleability is the ability of a material to undergo plastic deformation under compressive stresses such as NIL. FCC has more lattice planes on which deformation takes place. Au atoms slide past each other relatively easily and are comparatively more malleable than Al and Cu. The atomic plane of gold atoms is such that it allows for more sliding of its individual atoms over each other during deformation. This gives room for better deformation patterns compared to aluminum and copper. The malleability of metals is a combinatorial effect of several factors, including the metallic bond strength, ultimate tensile strength, Mohs hardness, and the resultant dislocation mobility of its atoms. Gold has the lowest Mohs hardness (2.5) and metallic bond strength of the three materials in this research and has the ability to undergo severe plastic deformation. Gold has shown deformation to sizes below sub-grain dimensions where other metals undergo fragmentation [48]. This can be attributed to the absence of oxide films due to its inert nature, which permits dislocations to escape without pile-up at grain boundaries. Though aluminum had lower ultimate tensile strength, it encounters higher dislocation pile-up at the grain boundaries [49,50], thereby increasing resistance to deformation and, thus, resulting in higher von Mises. Thus, in the case of gold, high-strain rate deformation results in continuous breakdown and reformation of sub-grains as dislocations are translated through the material, resulting in a comparatively lower von Mises stress.

The high-strain rate deformation during the NIL process induces compressive stresses, resulting in phase transformation from the native FCC lattice structures to BCC, HCP, and other spurious types. In this research, we investigated the effect of NIL mold penetration on geometric feature replication in gold, copper, and aluminum at ambient temperature (293 K). The deformation behavior was quantified based on von Mises stresses (through the six pressure tensors) and phase transformation (PTM method) induced, as shown in Table 2.

**Figure 5:**
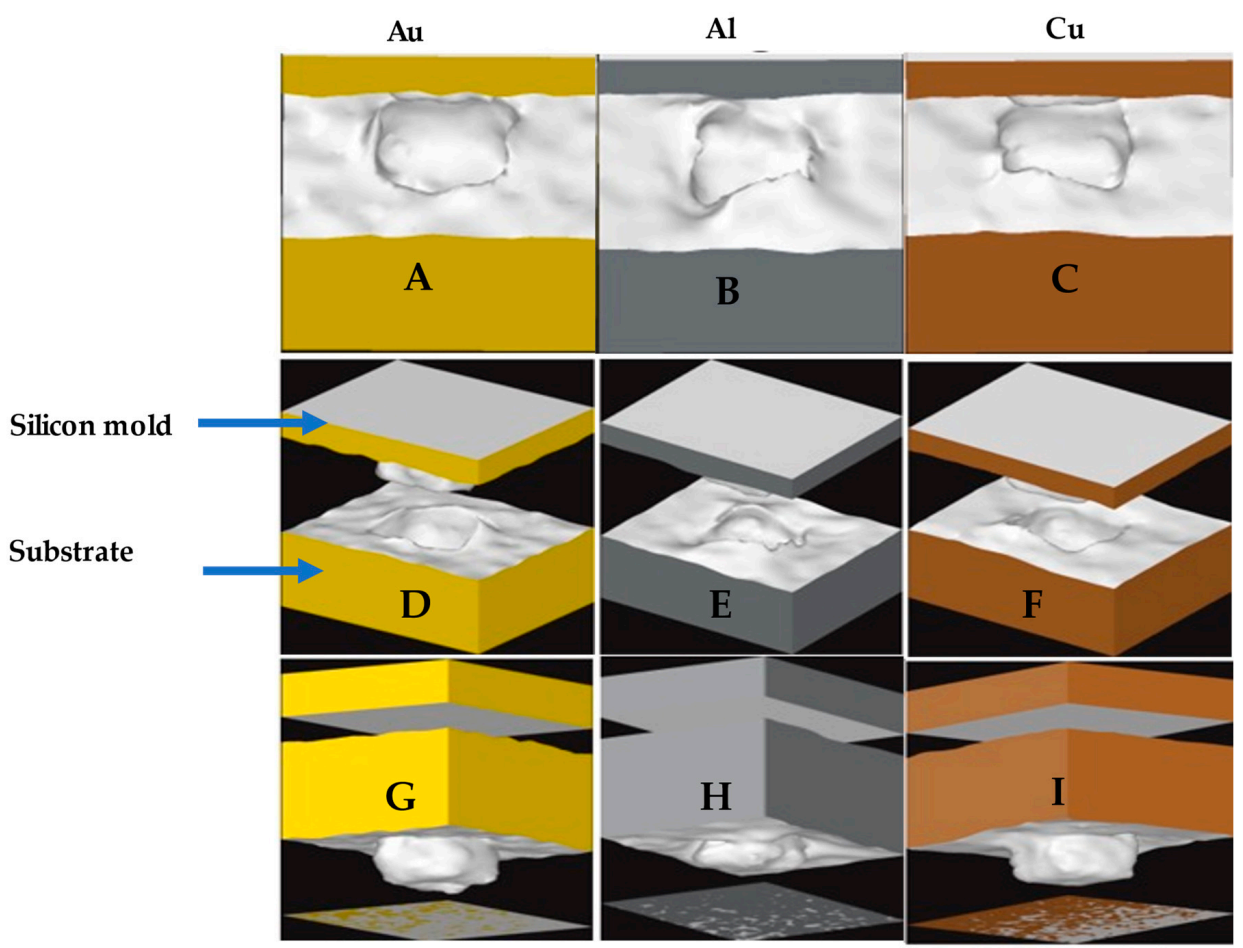
Deformation profile of gold, aluminum, and copper (after Demolding). (**A**–**C**) Top view of the deformation profile. (**D**–**F**) Orthogonal view of the deformation profile. (**G**–**I**) Inside-out view of the deformation profile.

After the thermal equilibration stage (Initialization), most lattices within the three materials have an FCC structure. The von Mises stresses after equilibration exist due to the realignment of atoms to attain ambient temperature (293 K). Cu (6.8 MPa) had the highest internal stresses, followed by Al (4.0 MPa) and Au (3.9 MPa). However, at the end of the molding stage, aluminum retained the highest FCC (65.7%) structure with minimal transformation to BCC (4.6%) lattice. The pile-up of dislocations at grain boundaries has been documented to induce strain-hardening and acts [51] as the pivotal reason that results in higher von Mises stresses (8.3 MPa) at the end of the molding phase. Typically, deformation-induced phase transformation occurs due to higher compressive forces within a confined volume, which converts the FCC to BCC lattice types along the Bain pathway [52]. This is evident in both Au and Cu lattice transformations at the end-of-molding stage wherein Au (32.2%) and Cu (25.7%) BCC lattices were formed with 5.4 MPa and 8.3 MPa von Mises stresses, respectively. This transformation can be attributed to the Bain path, which consists of the distortion of FCC substrate atoms in the x and y directions due to the movement of atoms away from the mold insertion area [53]. The periodic nature of the x and y lattices permits distortion towards phase transition. During the end-of-demolding stage, both Au and Cu regained partial FCC lattice structure with the relaxation of the compressive mold forces as evident based on lower von Mises stresses for Au (4.5 MPa) and Cu (7.7 MPa), respectively. During the demolding phase, FCC and BCC transitions occurred due to the dilation of atoms in the vertical (z-direction) for both Au (44.9%) and Cu (50.7%) materials, respectively. However, due to the strain-hardening behavior of Al, there were minimal changes to the lattice structures from the molding to demolding stages. Thus, lattice transformations have been responsible for cleaner NIL imprint profiles in both Au and Cu as compared to Al, as shown in Figure 5.

### 3.3. Deformation Behavior of Materials 

Figure 5 shows the deformation behavior of the final imprint after demolding obtained by generating surface meshes from the “construct surface mesh” modifier within OVITO. A, B, and C show the top view of the three materials after indentation and complete withdrawal. D, E, and F indicate the orthogonal view, including the silicon mold. In G, H, and I, the inside-out view of the indents has been presented. Gold exhibited a more precise replication of the NIL mold, followed by copper and aluminum. This observation aligns with the PTM images presented earlier, which showed a significant spring-back of Al atoms upon mold removal, as observed in Figure 5H. The inside-out view of the aluminum substrate after mold withdrawal revealed that the imprint was not that effective, and as a result, the mold replica was shorter in length (indicating lower cavity depth) as compared to that of gold and copper. The adhesion between the mold and the substrate and the release and material properties of the substrate have been reported in the literature as some of the factors that may affect the effectiveness of the nanoimprint lithography process. The irregularities of the indentation profile in aluminum can be attributed to its relatively lower ductility compared to copper and gold. Gold, being the most ductile material among the three, tends to flow and deform more uniformly, resulting in a smoother surface. Looking at the three profiles, copper and gold seem to have formed an almost rectangular slot imprint of the silicon mold after indentation. However, the aluminum material had a distorted imprint profile.

### 3.4. Spring-Back Phenomenon 

A positive spring-back in the horizontal direction indicates a contraction of the substrate shape (narrower slot or shorter cavity), whereas a negative spring-back indicates an expansion of the substrate shape (wider slot or longer cavity) in respective dimensions. This definition of positive and negative spring-back also holds in the vertical direction, with positive spring-back indicating a reduction in the depth of the imprint and negative spring-back showing an increase in indentation depth after mold withdrawal or retrieval(demolding).

**Figure 6:**
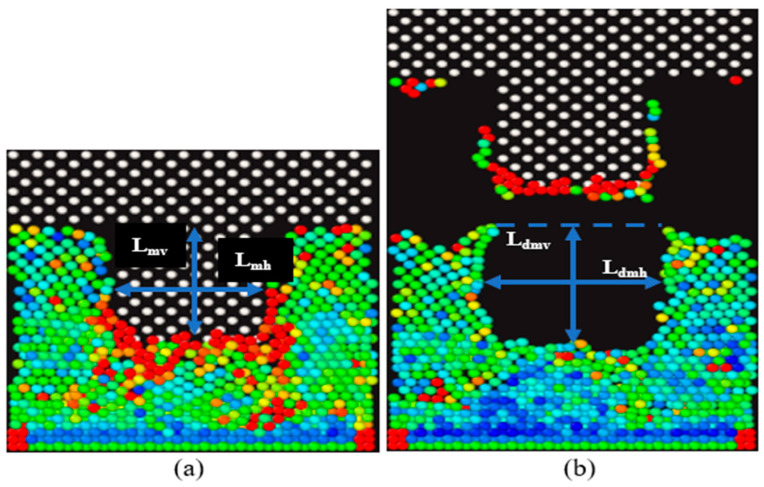
Illustration of cavity measurements for spring-back calculations during EOM and EOD stages. (**a**) Cavity measurement of spring-back for gold substrate in the vertical and horizontal direction at EOM. (**b**) Cavity measurement of spring-back for gold substrate in the vertical and horizontal direction at EOD.

We have provided an illustration to demonstrate the calculation of both horizontal and vertical spring-back, taking the deformation profile of gold as an example. Spring-back is calculated in each direction using Equation (3). Where % spring-back is the spring break value in the horizontal or vertical direction, *L_mold_* is the initial dimension during molding, and *L_demold_* is the final dimension after demolding, respectively. In Figure 6, multiple measurements (n = 5) were made in both horizontal and vertical dimensions during the molding stages, as shown in Figure 6a. Further, similar measurements were made after the demolding phase in Figure 6b. An average value was used for the (n = 5) measurements in each direction to ensure consistency in the deformation profiles of the substrates. These values were used to calculate the % spring-back in both orthogonal directions. We measured the cavity width for the molding and demolding stages and replicated five times (n = 5) to obtain measurements at different locations of the cavity using Image J2 software. We then computed the absolute average in the horizontal direction based on Equations (4a) and (4b).

(4a) $$L_{mold}\left(horizontal\right)=\frac{L_{mh1}+L_{mh2}+\cdots +L_{mh5}}{5}$$

(4b) $$L_{demold}\left(horizontal\right)=\frac{L_{dmh1}+L_{dmh2}+\cdots +L_{dmh5}}{5}$$

The spring-back is positive if the cavity shrinks after indentation, whereas it is negative if there is an expansion after the indentation. Similar calculations were conducted in the vertical direction based on Equations (5a) and (5b).

(5a) $$L_{mold}\left(vertical\right)=\frac{L_{mv1}+L_{mv2}+\cdots +L_{mv5}}{5}$$

(5b) $$L_{demold}\left(vertical\right)=\frac{L_{dmv1}+L_{dmv2}+\cdots +L_{dmv5}}{5}$$

Typically, the spring-back values in the vertical and horizontal directions should be minimal to achieve a high-quality imprint.

**Figure 7:**
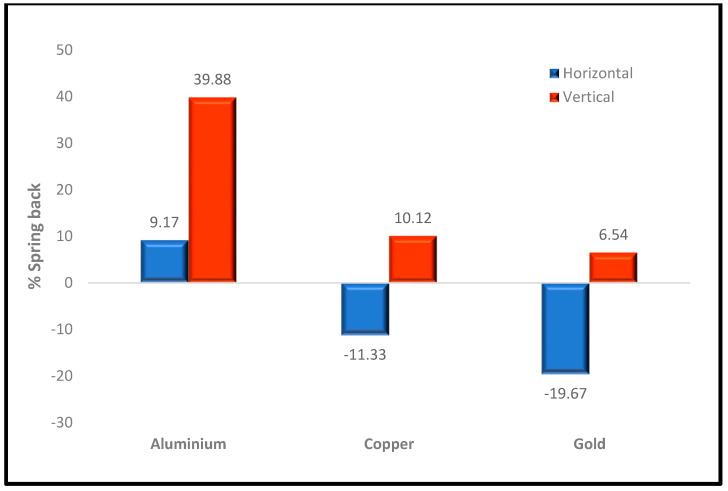
% Spring back for the three metal substrates imprinted with silicon mold profile at respective directions.

Figure 7 shows the percentage spring-back for the aluminum, copper, and gold substrates imprinted with the silicon mold at respective directions. This measure was implemented to quantify the quality of the imprint. The spring-back was measured in both vertical and horizontal directions. A high-quality imprint occurs when the variation of spring back is at the barest minimum in either or both directions.

The spring-back effect in the vertical direction was most prominent in the aluminum substrate (+39.88%), and the lowest was observed in gold (+6.58%). This finding is consistent with that obtained for PTM and von Mises stress for these materials, as presented in Figure 2 and Figure 4. Figure 2J and Figure 4G show that the indented cavity of the aluminum substrate reduced in the vertical direction, indicating a positive spring-back after mold retrieval. The aluminum substrate had the worst imprint quality after demolding, which is evidently shown in Figure 5H. The highest spring-back in the horizontal direction was observed in the gold substrate (−19.67%) and indicates an expansion in its mold profile. The copper substrate can be seen to have expanded in the horizontal direction with a negative spring-back of −11.33% and a positive spring-back of +10.12% in the vertical direction. Overall, copper and gold substrates have relatively cleaner imprints than aluminum. 

An important finding to also note in this research is that, despite the rough imprint profile of aluminum, it is able to retain 75% of its initial FCC structure at the end of the nanoimprint lithography process (as seen in Figure 3A,C). This is followed by copper and then gold at 63% and 55%, respectively. However, a higher spring back in aluminum resulted in the most distorted imprint.

## 4. Conclusions

The deformation characteristics of three distinct materials (aluminum, copper, and gold) have been systematically examined through molecular dynamics simulations within the context of Nanoimprint Lithography. These investigations were carried out by exploring the von Mises stress, lattice dislocation behavior, and material deformation across the aforementioned materials. During the entire nanoimprint phases, the copper substrate exhibited a notably higher von Mises stress (7.80 MPa) in comparison to both aluminum (4.95 MPa) and gold (4.68 MPa) substrates, respectively. Additionally, a considerable reduction in von Mises stresses was observed during the subsequent mold withdrawal stages (demolding) for all three materials. Among the three materials, aluminum displayed the most robust retention of its FCC structure (75%). It is noteworthy that aluminum manifested the highest percentage of vertical spring-back (+39.88%), whereas gold exhibited the highest percentage in the horizontal direction (−19.67%). Gold, followed by copper, had the cleanest imprints due to their highly malleable behavior. These outcomes provide a foundational framework for formulating design guidelines for nanoimprint molds for replication with other metallic materials.

## Figures and Tables

**Table 1:** Material properties of substrate and mold materials [47].

Element	Density (kg/m^3^)	Melting Point (K)	Lattice Constant (Å)	α^0^	β^0^	β^1^	β^2^	β^3^	t^(1)^	t^(2)^	t^(3)^	Ultimate Tensile Strength (MPa)
Silicon	2328	1687	5.431	4.87	4.4	5.5	5.5	5.5	3.13	4.47	1.80	170
Gold	19,300	1337	4.078	6.34	5.45	2.2	6	2.2	1.59	1.51	2.61	120
Aluminum	2710	933.5	4.046	4.61	2.1	2.2	6.0	2.2	1.78	2.21	8.01	90
Copper	8940	1358	3.597	5.11	3.63	2.2	6.0	2.2	3.14	2.49	2.95	210

**Table 2:** Lattice structure and von Mises stress data at different NIL stages.

Stages	Lattice Structure (%)	Copper	Aluminum	Gold
Equilibration	FCC	81	88.6	88.6
	BCC	0	0.5	0.4
	HCP	0.1	0.1	0.1
	OTHERS	18.9	10.8	10.9
	Stress (MPa)	6.8	4	3.9
EOM	FCC	42.4	65.7	42.6
	BCC	25.7	4.6	32.2
	HCP	14.9	15.6	12.2
	OTHERS	17	14.1	13
	Stress (MPa)	8.3	6.8	5.4
EOD	FCC	50.7	63.5	44.9
	BCC	9.4	4.2	23.3
	HCP	19.8	13.1	12.9
	OTHERS	20.1	19.2	18.9
	Stress (MPa)	7.7	4.6	4.5

## Data Availability

Data are contained within the article.
